# How to make better forensic decisions

**DOI:** 10.1073/pnas.2206567119

**Published:** 2022-09-13

**Authors:** Thomas D. Albright

**Affiliations:** ^a^The Salk Institute for Biological Studies, La Jolla, CA 92037

**Keywords:** signal detection, pattern comparison, forensic science

## Abstract

Much of forensic practice today involves human decisions about the origins of patterned sensory evidence, such as tool marks and fingerprints discovered at a crime scene. These decisions are made by trained observers who compare the evidential pattern to an exemplar pattern produced by the suspected source of the evidence. The decision consists of a determination as to whether the two patterns are similar enough to have come from the same source. Although forensic pattern comparison disciplines have for decades played a valued role in criminal investigation and prosecution, the extremely high personal and societal costs of failure—the conviction of innocent people—has elicited calls for caution and for the development of better practices. These calls have been heard by the scientific community involved in the study of human information processing, which has begun to offer much-needed perspectives on sensory measurement, discrimination, and classification in a forensic context. Here I draw from a well-established theoretical and empirical approach in sensory science to illustrate the vulnerabilities of contemporary pattern comparison disciplines and to suggest specific strategies for improvement.

Conscience does make cowards of us all.

*Hamlet* (1)

A large category of forensic evidence consists of patterned impressions, such as fingerprints, tire tracks, and tool marks that are created, often without intent or awareness, during the perpetration of a crime. The causal origins of these artifacts (the evidence) are assessed by trained observers who compare them to patterns that would have been produced under a hypothesized set of conditions (the model, or “exemplar”). By means of this “forensic pattern comparison” the observer makes a judgment about whether the percepts elicited by the evidence and the model are sufficiently similar to have common origin.

Despite much promise and long-standing public support, forensic practices that rely on human sensory judgments sometimes incriminate the wrong people (2, 3), with tragic personal and societal consequences. Many of the problems with forensic practice were considered in a landmark 2009 report from the National Academy of Sciences (NAS) (4), which identified numerous weaknesses and offered detailed recommendations for science-based reform. In 2015, President Obama asked the President’s Council of Advisors on Science and Technology (PCAST) to further evaluate needs within the forensic science community. PCAST observed, as did the NAS before it, that pattern comparison methods were particularly problematic (5).

In response to these concerns, there has been a growing movement to bring the modern sciences of human information processing—sensation, perception, and memory—to bear on the problem of forensic pattern comparison (e.g., refs. 6–13). In the following I lay out a conceptual and experimental approach drawn from these sciences that yields insights into the problems associated with pattern comparison disciplines and suggests strategies for reform.

## A Human Information Processing Approach

Much of human behavior is based on detection and measurement of stimuli in the sensory environment, followed by discrimination and classification of those stimuli for use in guiding choices and actions. While scientific understanding of these processes has advanced considerably in the past few decades, only recently has the knowledge gained been applied in efforts to improve forensic practice. The success of this newer approach is best exemplified by the problem of contextual bias, in which other sources of information, such as demographics or prior history of a suspect, unconsciously influence judgments of pattern similarity. Drawing from a rich well of concepts, methods, and data in sensory, cognitive, and neural sciences, which reveal how, why, and when such biases occur (e.g., refs. 14–16), significant progress has been made toward understanding and documenting the manifold sources of bias in forensic examination and implementing policies and procedures to overcome them (17–19).

In the following I bring the sciences of human information processing to bear on the related problem of stimulus classification under conditions of uncertainty in forensic pattern comparison disciplines. I maintain that the accuracy of these disciplines and their utility for the courts is thwarted by a failure to appreciate how the machine works, how people make decisions informed by sensory information. Building upon a well-established scientific foundation for understanding sensory decisions, I argue that there are simple ways to improve the quality and richness of information provided to the courts by forensic examiners, which will both enhance the fairness of criminal justice and heighten the credibility of pattern comparison disciplines. What follows is thus both tutorial and call to action, inspired by the highly successful introduction of concepts from decision sciences to other applications that rely critically upon information measurement and classification, such as medical diagnosis (20, 21), risk assessment (22), weather forecasting (23–25), and baggage security screening (26).

I begin with the simple assertion that there are two proximal factors that contribute to the accuracy of sensory classification decisions made by a human observer: 1) the properties of the stimuli received by the senses and 2) the operating characteristics of the observer. Observer operating characteristics, in turn, have two attributes that govern performance: 1) sensitivity for relevant properties of sensory stimuli and 2) selectivity of the criteria used for stimulus classification. This characterization places the forensic classification problem squarely in the domain of signal detection theory (27), which affords a principled understanding of sensory decisions and insights on how to improve their utility for the courts.

## Forensic Classification as a Signal Detection Problem

**Fig. 1.**
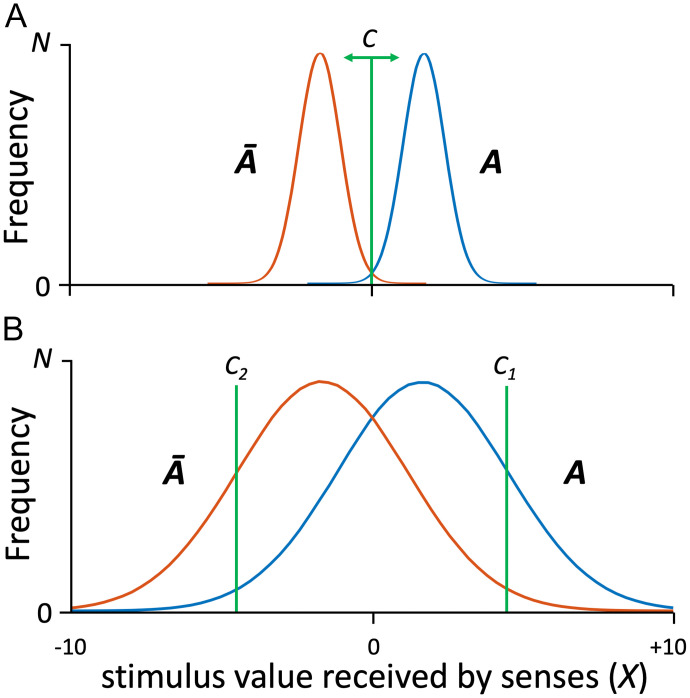
Formal description of pattern classification problem in forensic science. The task of the forensic examiner is to determine whether a given sensory stimulus (a piece of forensic evidence) of value *X* (abscissa) originates from source *A* or *Ā*. The examiner makes this determination by visually comparing the evidence to an exemplar known to have been produced by source *A*. Performance depends on properties of the stimuli received and the sensitivity and selectivity of the examiner. (*A*) Curves plot frequencies of different values of *X* that result from sources *A* (blue) and *Ā* (red). Patterns received by senses are high-quality, each manifesting only a small range of values. Decision criterion *C* can easily be placed at a point (*X* = 0) that yields near-perfect classification of *X* as *A* vs. *Ā*. (*B*) Patterns impressed by objects *A* and *Ā* are distorted by sources of noise, such that each frequency distribution manifests a broad and overlapping range of stimulus values. No single criterion *C* will yield clean classification. *C_1_* is stringent, yielding a high rate of correct identifications of *A* but missing many instances of *A*. Criterion *C_2_* is loose, identifying nearly all correct instances of *A,* but also admitting many incorrect instances.

Forensic stimuli received by the senses often consist of patterned impressions found on surfaces in a crime scene, caused by objects that have come in contact with those surfaces. (Here the focus is on visual patterns, which are frequent in the forensic context, with recognition that the same logic applies to information received by other sensory modalities.) These patterns commonly vary along multiple dimensions, such as luminance, chrominance, texture, size, and shape. To simplify exposition, imagine that we collapse this sensory variation to a single dimension *X*, as illustrated in Fig. 1. In this example, visual patterns created by object *A* manifest values of *X* that appear variously with frequencies indicated by the blue curve in Fig. 1*A*. This variation reflects noise, some of which results from creation of the pattern (e.g., occlusions and smears) or subsequent deterioration, and some (e.g., optical and neuronal noise) reflects properties of the observer. Similarly, patterns created by objects *Ā* (*not-A*) have values of *X* that appear with frequencies indicated by the red curve in Fig. 1*A*. The observer’s task is to decide whether a given value of *X* (the stimulus value received by the senses) was caused by *A* or *Ā*. In practice, this decision about the source of evidence *X* is made by comparing it to an instance of *X* that is known to have been produced by object *A*, i.e., an exemplar of *A*. By this means, the decision about source is reduced to a decision about the apparent similarity of evidence and exemplar, which is where the fine sensitivity of the human visual system comes into play. The cutoff value of *X* that distinguishes an observer’s classification of evidence as originating from *A* vs. *Ā* (i.e., evidence and exemplar as matching vs. nonmatching pairs) is called the “decision criterion.”*

In the case illustrated in Fig. 1*A*, patterns originating from objects *A* and *Ā* are both of high signal quality (low noise variance), such that the *A* and *Ā* frequency distributions barely overlap. A decision criterion of *X* = 0 (green line, Fig. 1*A*) is ideal, since values of *X* that exceed zero are almost certainly visual patterns caused by *A*, and values less than zero are almost certainly caused by *Ā*. This scenario provides a reference point of excellent decision-making, but there are many conditions in the real world in which different objects cause highly overlapping distributions of sensations (Fig. 1*B*). Under these conditions, no single decision criterion will uniquely differentiate origin from *A* vs. *Ā*. In some cases, sensory evidence *X* caused by *A* will be classified as originating from *Ā*, and vice versa. If we were to place the decision criterion at *X* = 0 in Fig. 1*B*, as we did for Fig. 1*A*, the probability of correctly deciding that a visual pattern was produced by *A* would be 0.72, but the probability of incorrectly making that same decision is a worrisome 0.28.^†^

The decision criterion we actually apply under the more difficult conditions of Fig. 1*B* will depend upon how we prioritize the outcomes. To appreciate this, consider the situation in which vibration caused by a cell phone (object *A*, manifested as a tactile pattern in this case) must be distinguished from subtle tactile stimuli of other causes (objects *Ā*). If an expected phone call is important, we might lower the cutoff value of *X* that serves as our decision criterion for responding (*C_2_* in Fig. 1*B*), but in doing so we risk interruption by many sensory events that are not phone calls. Conversely, if we dislike interruptions we might raise our criterion (*C_1_* in Fig. 1*B*). Doing so makes it more likely that the stimulus we respond to is in fact a phone call, but we miss many other calls in the process.

In this signal detection framework, the ability to overcome the overlap of patterned sensations from different objects is a measure of the discriminability, or sensitivity, of the observer in the presence of noise (27). The priority given to different outcomes determines the selectivity of the observer’s criterion for deciding between different classifications of the sensory evidence. What we end up with is a decision that is either correct (*X_A_* classified as originating from *A*, *X_Ā_* classified as *Ā*) or incorrect (*X_A_* classified as *Ā*, *X_Ā_* classified as *A*). If the recipient of a decision knows 1) the frequency distributions of patterned sensations caused by the relevant source objects, 2) the sensitivity of the measurement device (the observer, in this case), and 3) the selectivity of the decision criterion, the recipient can infer the probability that the decision is correct. In engineered binary classification systems, such as smartphone fingerprint detectors, these properties are partially known, which means that the manufacturer can offer some assurances about accuracy. In the case of human observers, however, this information is harder to come by, which means that the recipient of a single classification decision may not know whether it was the product of high sensitivity and a highly selective criterion or poor sensitivity and a loose criterion. That difference matters, of course, because the former is much more likely to be a correct decision.

In the following, I show that this signal detection framework reveals conceptual and procedural flaws in current forensic practice and suggests empirically testable approaches that could improve judicial outcomes. I focus specifically on the problem of forensic firearms examination—mainly to make the exposition concrete—but stress that the principles and inventions have relevance to all forensic disciplines that involve measurement and classification of sensory evidence by human observers.

## Forensic Firearms Analysis

Forensic firearms examination is a subdiscipline of tool mark forensics. The practice is based on the fact that when a round of ammunition is fired, the machined hard steel components (“tools”) of firearms, such as the breech face, firing pin, chamber, and barrel, make patterned impressions under high pressure in the softer metals of the cartridge case, primer, and bullet. The underlying premise is that the resulting patterns are unique to a gun—like fingerprints unique to a person—and thus it should be possible to determine whether a pattern is consistent with origin from a particular gun, perhaps one owned by a suspect. In the following discussions I focus on the cartridge case identification problem (bullets are sometimes mushed or fragmented upon impact).

Forensic firearms examination dates to the 19th century and has been in common practice—and accepted as evidence in US and state courts—for over 100 y (28). The principle behind this practice is that an observer’s visual system can measure and compare forensic and known source samples through microscopic examination and decide whether they are similar enough to have come from the same firearm (29). This principle was formalized and codified 30 y ago by the professional organization known as the Association of Firearm and Tool Mark Examiners (AFTE), in their “theory of identification as it relates to toolmarks” (30). The essence of this theory is that it “enables opinions of common origin to be made when the unique surface contours of two toolmarks are in ‘sufficient agreement.’” At first blush, “sufficient agreement” seems a poorly defined criterion, but the “theory” goes on to state that “Agreement is significant when the agreement in individual characteristics exceeds the best agreement demonstrated between toolmarks known to have been produced by different tools and is consistent with agreement demonstrated by toolmarks known to have been produced by the same tool.” By this statement, the criterion for a life shaping decision is defined in relative terms, based on the examiner’s experience-dependent inferences about the probability distributions of sensory patterns.

In common practice, pattern source decisions in firearms analysis are based on three types of pattern variation, known as class, subclass, and individual characteristics. Class characteristics are typically those associated with specific firearm manufacturers, such as the caliber of the cartridge, or impressions created by a proprietary cartridge ejector mechanism (the machined part of the gun that tosses the cartridge out after it has been extracted from the chamber). Subclass characteristics are those unique to gun parts produced by a specific manufacturing device (e.g., a lathe) within a class, such as incidental machining marks on a breech face that appear similarly on all guns produced by that same device. Individual characteristics are those unique to a specific gun, as would result from incidental machining marks on the combination of manufactured parts and actions of that gun. (Subclass characteristics are effectively a form of camouflage that confound an observer’s ability to detect individual characteristics.)

**Fig. 2.**
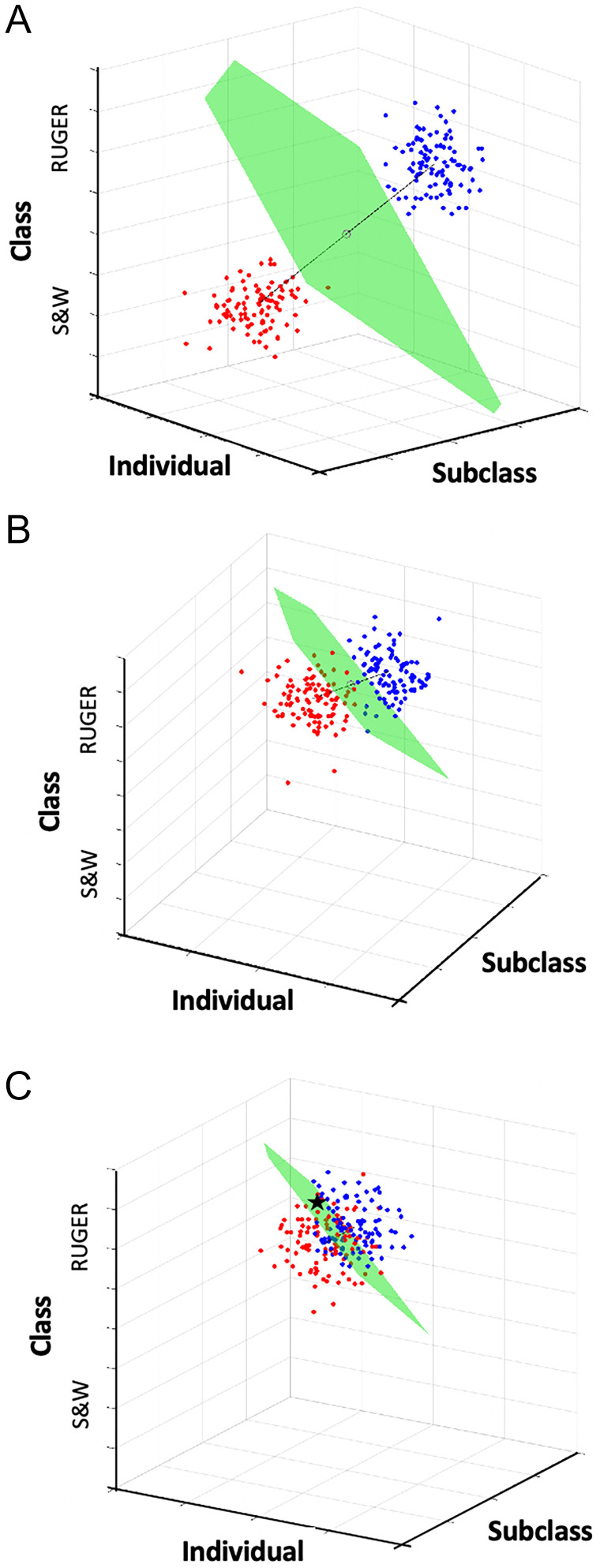
Three-dimensional illustrations of forensic firearms classification problem. Forensic evaluation of firearms evidence (cartridge cases and bullets) is based on three known sources of variation (“characteristics”) in the sensory evidence. Class characteristics refer to features that are associated with specific manufacturers or models of guns. Subclass characteristics are patterned impressions common to a machining device within a class. Individual characteristics are patterned impressions unique to a specific gun. (*A*) Three-dimensional scatterplots of hypothetical sensory values produced by two guns. Blue dots are values from gun *A* (Ruger), red dots are from gun(s) *Ā* (S&W). Noise variance for each gun is modeled as a 3D normal distribution. Thin black line connects means of the two distributions. Green plane is criterion that most cleanly separates the frequency distributions. (*B*) In this case the two guns are same class and manifest partially overlapping distributions of subclass and individual characteristics. Green plane is midpoint decision criterion, which reveals that the two distributions cannot be cleanly separated. (*C*) In this case the distributions are highly overlapping along all three dimensions, creating a significant challenge for a binary classifier. Black star represents pattern “produced” by gun *A*, but easily confused with gun(s) *Ā*.

The firearm sensory comparison problem follows the general framework illustrated in Fig. 1. Because there are three “systematic” types of pattern variation (class, subclass, and individual) in the sensory evidence received, in addition to stochastic variation (noise), it is helpful to conceptualize this signal detection problem at the outset by viewing it graphically in three-dimensional (3D) space. Fig. 2*A* contains two 3D scatter plots, which represent hypothetical frequency distributions of cartridge evidence produced by guns of two classes, Ruger (gun *A*, blue dots; the suspected source of the evidence) and S&W (gun *Ā*, red dots). As expected, class, subclass, and individual characteristics for the two guns have no overlap, which suggests a clean decision criterion (green plane in Fig. 2*A*) for identification vs. elimination. Fig. 2*B* shows a case in which two hypothetical frequency distributions (blue and red dots) are consistent with two guns of the same class (Ruger). Here, subclass and individual characteristics are partially overlapping, increasing the difficulty of the classification problem. Finally, Fig. 2*C* shows a case in which class and subclass distributions are largely overlapping, consistent with two Rugers sequentially manufactured using the same equipment. Individual characteristics are also highly overlapping. Accurate classification of evidence based on these distributions is extremely difficult, since no decision criterion provides clean separation. For example, the patterned evidence corresponding to the black star in Fig. 2*C* was “produced” by gun *A*, but that evidence is also consistent with production by gun(s) *Ā*.

As for other forensic pattern comparison disciplines (e.g., ref. 31 ), the firearms community attempts to solve this decision criterion problem by offering multiple classification options. According to the “AFTE Range of Conclusions” (32), in addition to “identification” and “elimination,” examiners can report “inconclusive” or “unsuitable.” Unsuitable means that there is insufficient information to measure, much less compare. The more interesting “inconclusive” classification is a statement about the examiner’s inability to confidently establish a decision criterion for identification vs. elimination. Given the perilous sequalae of a forensic classification error, the natural human tendency is to default on difficult problems. Doing so, however, creates a different set of concerns, which have tied the field in knots and raise serious questions about the utility of firearms evidence (33–37).

## The Flawed Logic of “Inconclusive” Responses

**Fig. 3.**
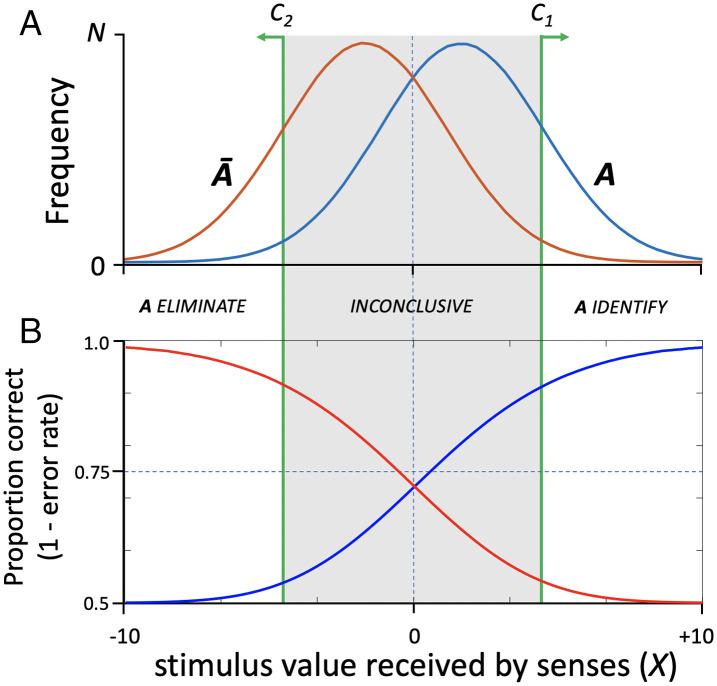
Effect of “inconclusive” option on quality of validation data. (*A*) Frequency distributions of *X* originating from objects *A* and *Ā*. Use of two decision criteria (*C_1_* and *C_2_*) affords clean classification of some values of *X* as originating with *A* vs. *Ā*, but all values between those decision criteria (gray shading) are deemed “inconclusive.” (*B*) Modeled performance as a function of *X* given hypothetical frequency distributions in *A*. Accuracy for *A* decisions (proportion correct identifications) is plotted in blue. Accuracy for *Ā* decisions (proportion correct eliminations) is plotted in red. The upper extents of blue and red curves summarize maximum potential for correct classification. Use of the inconclusive option, however, prevents quantification of performance for a meaningful set of stimuli and increases performance estimates for the remaining stimuli.

Considered in signal detection terms, the use of an inconclusive option forces the adoption of two decision criteria. To simply illustrate the consequences of doing so, we can collapse the 3D space of Fig. 2*C* onto the 1D form used in Fig. 1. In Fig. 3*A*, the upper and lower decision criteria (*C_1_* and *C_2_*) are indicated by green lines. Values of *X* that exceed the upper bound can be reliably classified as *A* (identification). Similarly, those below the lower bound can be reliably classified as *Ā* (elimination). The forensic examiner defaults on everything between the two decision criteria (shaded region of Fig. 3*A*); that is, all cases in which the probability of correct classification is significantly limited by noise are deemed inconclusive.

There are problems associated with this practice. First, use of the inconclusive option means that the forensic method may have little utility for large numbers of cases, depending on the distribution of sensory evidence, sensitivity of the examiner, and the examiner’s placement of the two decision criteria. Second, the practice precludes assessment of the performance of forensic examiners for evidence bounded by the two decision criteria, which would be enormously valuable for establishing the true operating characteristics of forensic examiners and error rates of the discipline. We can, however, model what performance would look like generally, which highlights limitations of the inconclusive approach. Fig. 3*B* is a plot of a modeled examiner’s classification performance given the stimulus distributions in Fig. 3*A*. The blue curve plots proportion of correct identifications of *A* (true positive decisions, TP), which range from slightly greater than chance probability (0.5) to nearly 1.0.^‡^ The red curve plots proportion of correct eliminations (true negative decisions, TN). Performance curves for these two types of correct decisions intersect in the middle, with probability of a correct identification or elimination decision thus ranging from 0.72 to 0.99.

Strategic placement of decision criteria for identification and elimination makes average error rate unsurprisingly low outside of the inconclusive zone, but the rate more than doubles when that zone is included (shaded region of Fig. 3*B*). We know that this is true here because it is the product of a deterministic model. In real forensic practice, however, the frequency distributions of sensory evidence may not be fully known. Moreover, the quantitative relationship between those distributions and the examiner’s placement of decision criteria that bound the inconclusive zone is inscrutable to anyone receiving the decision (e.g., the trier of fact). To make matters worse, those criteria are potentially unstable across time and examiners, which likely accounts for substantial variability in reported rates of inconclusive responses across existing validation studies (34). Greater separation of *C_1_* and *C_2_* will naturally lead to an illusion of improved examiner performance, since it excludes from consideration more cases in which the probability that evidence *X* was produced by gun *A* is less distinct from the probability that *X* was produced by gun(s) *Ā*. For this reason, reports of low error rates based solely on identification and elimination decisions, with inconclusive judgments uncounted (5, 38), are uninformative products of circular reasoning: Examiners simply do well on classification problems that they find easier to judge.

Reformist efforts to include inconclusive responses in calculation of error rate lead to conceptual conundrums: Is inconclusive an error (a missed identification or a missed elimination) (34, 39, 40)? Or, as some have argued (41), isn’t inconclusive a correct response by exclusion of error?—because it cannot be counted as examiner error if the sensory information does not support identification or elimination? In other studies, the disposition of inconclusive responses is less a matter of principle: They are simply included in the denominator but not the numerator when calculating error rates (42). None of this epistemological floundering provides any real insight into actual error rate, but it never fails to fuel debate about the validity of forensic practice.

## Comprehensive Assessment of Forensic Examiner Performance

Signal detection theory suggests a better approach to the forensic pattern comparison problem. To illustrate, recall that under conditions such as those shown in Fig. 3, all decisions about source are inconclusive to some degree, quantified as a continuous function that describes the probability of a correct decision over values of evidence *X*. Traditional measures of error rate for identification and elimination decisions are thus probabilistic. Stimulus comparisons that are deemed inconclusive also have measurable probabilities of error. It’s not that these latter comparisons cannot be performed. Examiners simply choose not to perform them because they—in a thinly veiled invasion of the province of the jury—have reckoned the probability of a correct response to be insufficient for a finding of fact.

I propose here an alternative design of the firearms pattern comparison task, rooted in well-established principles of signal detection and inspired by recent pioneering work on the problem of eyewitness identification (7, 43, 44). In this task design, a version of which was used in a recent study of fingerprint examiner expertise (9), the examiner is required to make a choice between identification and elimination for every piece of evidence encountered—that is, for the full range of sensory evidence *X*—regardless of how difficult the decision may be. To illustrate, consider a hypothetical validation study that requires examiners to make forced choices between identification and elimination, using samples of evidence *X* drawn at random from the *A* and *Ā* frequency distributions shown in Fig. 3*A*. We draw 1,000 samples and find the true positive (TP) and false positive (FP) responses from this study to be 680 and 320, yielding an identification error rate of 32%. The forensic method is clearly not perfect, on average, but this overall percentage is as woefully uninformative of the nuances of examiner performance as is the low average error rates computed using an inconclusive option.

What is needed is an assessment of performance as a function of the criterion used to make the decision. While identification accuracy declines as an observer’s criterion moves from strongly to weakly selective (27), the criterion used by an observer for any given decision is an internal property of the observer and difficult to determine objectively. However, growing evidence from studies of eyewitness identification demonstrates that under “pristine” conditions^§^ an expression of confidence in the initial identification decision is highly predictive of the accuracy of that decision (43–45). These findings support the claim that confidence can serve as an accessible proxy for the observer’s decision criterion, which comports with the intuition that selective deciders are generally more confident in their decisions.

**Fig. 4.**
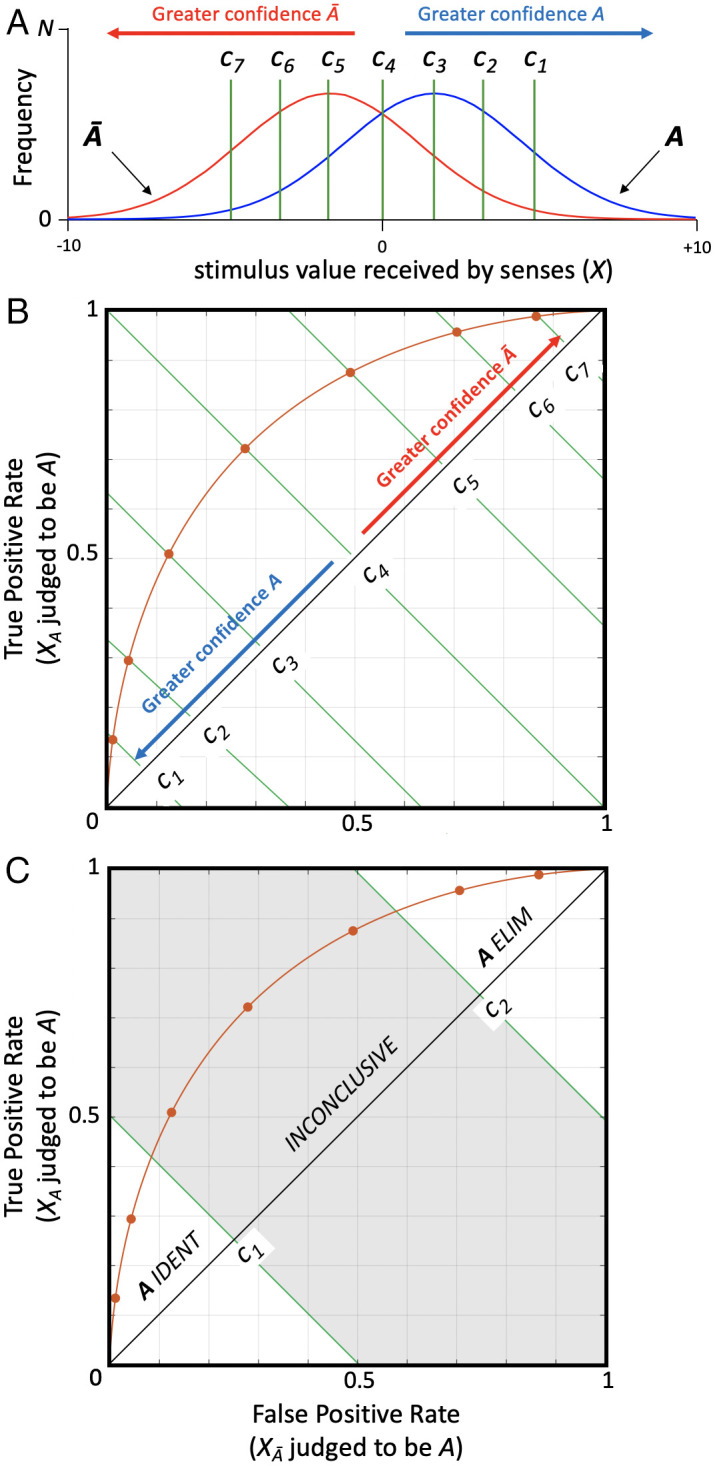
Classification decisions as a function of confidence. (*A*) Seven decision criteria (*C_1_*–*C_7_*) define eight levels of confidence in an *A* decision. Confidence levels range from strongly confident in an *A* decision (right end of abscissa) to weakly confident in an *A* decision (i.e., strongly confident in a *Ā* decision; left end of abscissa). For each confidence level, number of correct *A* decisions (true positives, TP) reflect area under the blue curve to the right of the confidence level indicator, and number of incorrect *A* decisions (false positives, FP) reflect area under red curve to right of the confidence level indicator. (*B*) Data points on red ROC curve plot TP rate vs. FP rate for the seven different criteria (*C_1_*–*C_7_*) defined by confidence levels. Confidence levels range along the positive diagonal from strongly confident in *A* (lower left) to weakly confident in *A* (upper right). Lines along negative diagonal represent criteria for the eight confidence categories. The ROC comprehensively summarizes examiner performance on classification of *X* as originating from object *A* vs. *Ā*. (*C*) Shaded region superimposed on ROC from *B* represents examiner performance not measured when inconclusive responses are permitted.

Building on this relationship between confidence and accuracy, suppose we require forensic examiners to render an 8-point confidence judgment with every forced choice, ranging from highly confident of an *A* decision (identification) to highly confident of *Ā* (elimination). Fig. 4*A* illustrates how this works with respect to the probability distributions for patterns being examined. There are now seven criteria (*C_1_-C_7_*) that divide eight levels of expressed confidence—*C_1_* being the most stringent confidence criterion and *C_7_* the most liberal—but in all cases an *A* vs. *Ā* decision is required. Inconclusive is not an option. By plotting proportion of correct identifications of *A* (TP) vs. incorrect identifications of *A* (FP) for each confidence criterion in Fig. 4*A*, we obtain the receiver operating characteristic (ROC) curve in Fig. 4*B*. When the examiner is strongly confident in an *A* decision (lower left quadrant of ROC), TP rate for *A* far exceeds FP rate for *A*. By contrast, when the examiner is weakly confident in an *A* decision (upper right quadrant of ROC), FP rate for *A* is nearly as large as TP rate for *A*. The shaded region in Fig. 4*C* indicates the wealth of performance data lost by permitting inconclusive responses.

The analysis illustrated in Fig. 4*B* offers benefits for both forensic validation studies and actual casework. First, the ROC is a complete summary of performance. If the foregoing procedures are applied to a population of forensic examiners in a validation study, the resulting ROC will quantify the expertise of the discipline as the overall ability to discriminate between sensory evidence originating from objects *A* and *Ā* (quantified by the area under the curve, AUC). Moreover, it captures performance as a function of the decision criterion used by different examiners (the selectivity of the criterion varies along the positive diagonal). This account of performance is superior to the single value error rate that forensic validation studies traditionally seek, in large part because it enables the recipient of a forensic decision to untangle the contributions of examiner sensitivity and the selectivity of the criteron used to make the decision.

**Fig. 5.**
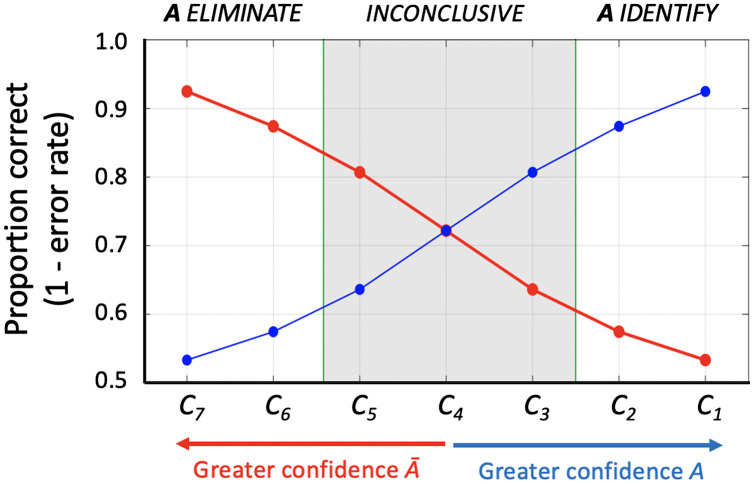
Confidence–accuracy curve. Plot of accuracy as a function of confidence derived from the model summarized in Fig. 4. Blue curve plots accuracy of *A* identification decisions [TP/(TP+FP)], which increases as confidence in those decisions rises. Conversely, red curve plots accuracy of *A* elimination decisions [TN/(TN+FN)], which increases as confidence in *A* declines and confidence in *Ā* rises.

Second, it is a simple matter to calculate error rates for the confidence-based decision criteria. Fig. 5 illustrates how accuracy (1 − error rate) can be derived from empirically determined rates of true and false positive decisions, as well as true and false negative decisions. The blue curve shows accuracy vs. confidence for identifications of *A*. Similarly, the red curve shows accuracy for eliminations of *A*. Although probability of a correct identification decision ranges from 0.53 to 0.93 in this example, overall accuracy for identifications and eliminations varies between 0.72 and 0.93 (assuming that the observer chooses identification vs. elimination based on the ratio of inferred likelihoods that evidence *X* would be expected given source *A* vs source *Ā*). A validity test conducted in this way is uncompromised by selective default on decisions and is thus a complete summary of the performance of the examiner. The shaded region of the accuracy plot indicates performance data lost by allowing the examiner to opt out with inconclusive responses, which further highlights the spurious nature of low error rate claims from traditional validation studies.

## Application to Real Casework?

A small number of studies with known source samples have successfully employed elements of the signal detection approach I advocate here, including the aforementioned study of fingerprint examiners (9), a recent study of firearms examiners that used examiner estimates of likelihood ratios as proxy for decision criteria (46), and an earlier investigation of firearms examiners using limited reports of confidence (6). This evidence provides novel insight into examiner performance on known-source tasks. When considered together with the foregoing analysis, it may be easy to concede the benefits of the signal detection approach for forensic task validation. On the other hand, it is fair to question whether the approach has any relevance to forensic decisions in real casework. Inconclusive responses are routinely permitted—many argue unavoidable—in casework because the consequences of error can be profound. Some argue further that a validation test that does not include inconclusive responses (one that does not match conditions of the real world) lacks ecological validity. In response to these arguments, I suggest that the signal detection approach presented here is worthy of consideration for real criminal casework because it may supply what the trier of fact truly needs to know: the examiner’s decision, together with an estimate of the probability that the decision is correct.

But how, one might fairly ask, do we obtain an estimate of the probability that a forensic decision is correct, when ground truth is unknown? The answer lies in the relationship between confidence and accuracy. The validation method described above, when applied to a given examiner in a known-source task, yields a measure of accuracy as a function of the examiner’s confidence. I submit that the predictive confidence–accuracy relationship determined by this means (Fig. 5) can serve as a “personal equation” (47, 48) for decoding that same examiner’s confidence judgments into estimates of accuracy in real casework, which the trier of fact can use to make well-informed decisions. This is an eminently testable hypothesis, but it raises important questions at the outset about the ability of an examiner’s confidence–accuracy relationship to generalize robustly across different conditions of sensory stimulation.

## Robustness of the Personal Equation in the Face of External Sensory Noise?

The confidence–accuracy relationship assessed for any examiner is necessarily based, in part, on learned information about the frequency distributions of the relevant sensory stimuli. This naturally includes information about central tendencies of the stimuli being compared. It also reflects implicit knowledge of noise of two general types: “receiver noise,” which is imposed by the examiner’s sensory apparatus (due to optical, neuronal, and attentional infidelity), and “emitter noise,” or stochastic variations in patterned impressions from a common source. We can reasonably assume that receiver noise for a given examiner is stable; not indefinitely, but at least over short windows of time in the same context. (Receiver noise may vary between examiners; all else being equal, some examiners have greater sensitivity than others.) Emitter noise is also likely to be stable for a given firearm. Patterned impressions on cartridge cases that have recently been fired will manifest noise of stable variance caused by the explosion in the chamber of the firearm. However, spent cartridges that have subsequently been exposed to corrosive substances or mechanical forces (“weathering”) may have additional “external” noise, thus making them more difficult to classify by the same examiner and perhaps causing the examiner’s confidence–accuracy decoder to fail.

**Fig. 6.**
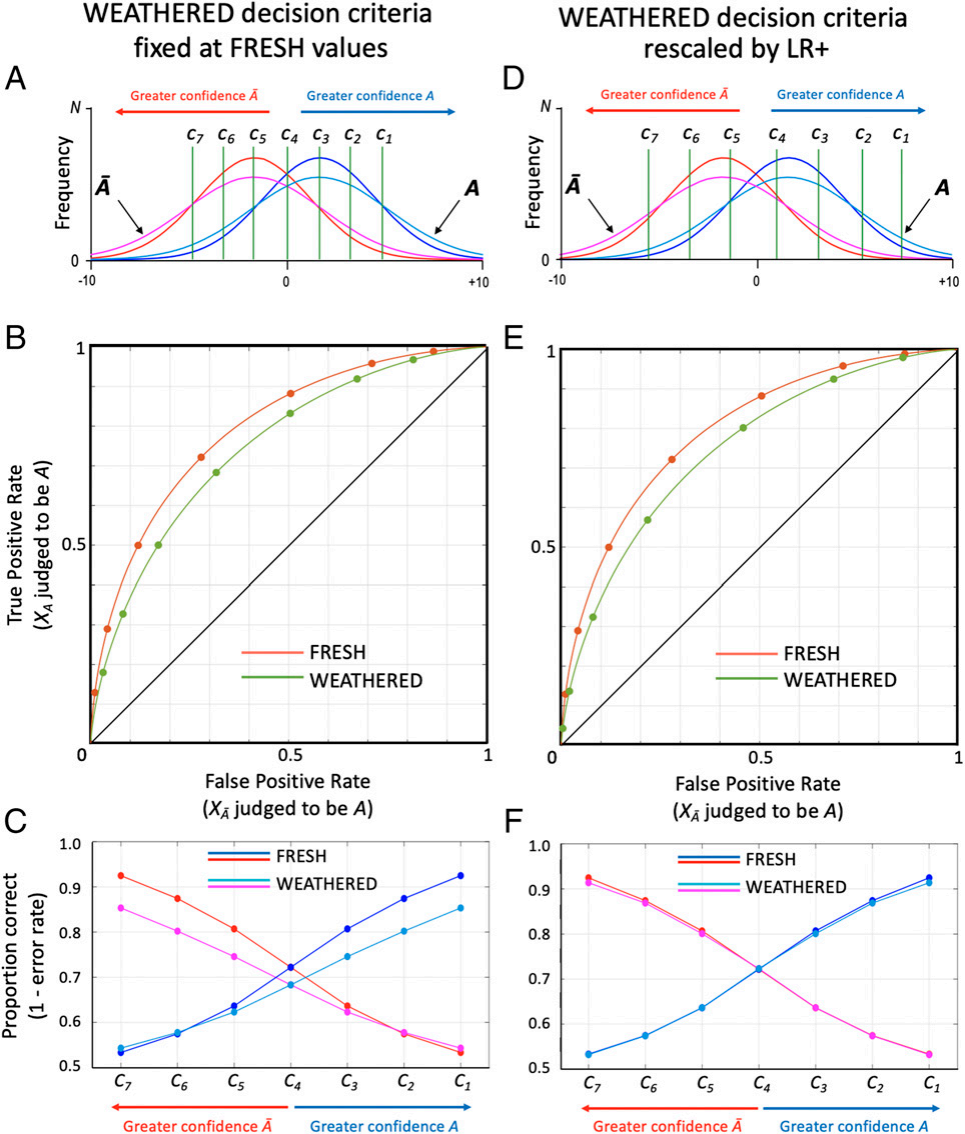
Rescaling of expressed confidence to accommodate task difficulty. (*A*–*C*) Scenario in which decision criteria are fixed to specific values of *X*; (*D*–*F*) scenario in which decision criteria are rescaled to track likelihood ratios across changes in task difficulty. (*A*) Plot of frequency distributions for “fresh” (blue and red) and “weathered” (teal and magenta) conditions. Green vertical lines (*C_1_–C_7_*) identify criterion values of *X* that define eight confidence levels for classification of *X*. (*B*) ROC curves based on frequency distributions in *A* for fresh and weathered conditions. Data points for both curves are derived from criteria specified in *A*. (*C*) Confidence–accuracy plots for fresh and weathered conditions, based on data points in *B*. Blue/teal curves represent accuracy of *A* identification decisions; red/magenta curves represent accuracy of *A* elimination decisions. Use of same (stimulus defined) confidence criteria for both conditions results in significant loss of accuracy for weathered condition, relative to fresh. (*D*) Frequency distributions for fresh and weathered evidence conditions. Confidence levels have been shifted rightward in an effort to equilibrate *A*/*Ā* likelihoods across changes in task difficulty. (*E*) ROCs for fresh and weathered frequency distributions. Data points for fresh condition are same as in *B*; those for weathered condition are based on rescaled confidence criteria from *D*. (*F*) Confidence–accuracy plots for fresh and weathered conditions, based on data points in *E*. Use of confidence criteria that have been rescaled to track likelihood ratios equates accuracy for fresh and weathered condition.

This potential for failure is illustrated graphically in the left column of Fig. 6. Fig. 6*A* contains a reproduction of the stimulus frequency distributions and confidence criteria from Fig. 4*A*, which represent a “fresh” condition of recently ejected cartridges. Superimposed upon the fresh stimulus distributions are distributions for a weathered condition, which is characterized by increased variance (relative to fresh) of patterned evidence *X* from objects *A* and *Ā*. Fig. 6*B* plots ROCs for both fresh (orange) and weathered (olive) conditions, derived using decision criteria fixed to the same values of *X*. These ROCs reveal the expected loss of discriminability for the weathered condition relative to fresh, manifested as smaller area under the curve. The corresponding confidence–accuracy curve in Fig. 6*C* reveals something more interesting: a significant decline in accuracy for the weathered condition. The confidence–accuracy decoder has failed. Empirically determined accuracy from a fresh condition validation study may serve as a valuable predictor of accuracy for a fresh condition in the real world, but this analysis suggests that it could be a poor predictor of accuracy for a weathered condition in the real world.

The only way that the confidence–accuracy decoder could generalize across conditions of different variance is if there were a rescaling of the observer’s decision criteria. But what is the correct type of rescaling, and how might it be imposed? The answer to this question has been a dominant thread in the scientific study of recognition memory for nearly 50 y. A well-known regularity in recognition memory performance is the “mirror effect,” which is (in essence) the finding that FP recognition rates mirror TP rates, regardless of the difficulty of the discrimination conditions (49, 50). This effect cannot be produced by fixing decision criteria at the same values of *X* (green vertical lines in Fig. 6*A*), because doing so necessarily alters the TP rate relative to the FP rate as task difficulty changes. This is precisely what happens in the fixed decision criteria model of Fig. 6 *A*–*C*, as manifested by changes in accuracy (Fig. 6*C*).

The mirror effect can be explained, instead, by likelihood-based scaling of the decision axis, which has become a standard feature of models of recognition memory (e.g., refs. 51–55). I suggest that the forensic examiner’s decision criteria for pattern comparison tasks may be similarly rescaled according to experience-based inferences about the likelihood that sensory value *X* originated from *A* vs. *Ā*. By this hypothesis, the observer adopts criteria that track likelihood ratios across different task difficulty conditions (56, 57).^#^

This likelihood-based rescaling proposal is illustrated in the right column of Fig. 6. Fig. 6*D* again plots stimulus frequency distributions for fresh and weathered conditions. In this example the decision criteria for the fresh condition are at the same initial values shown in Fig. 6*A*. The decision criteria for the weathered condition, shown in Fig. 6*D*, have been rescaled based on the shifted likelihood ratios associated with the more difficult task conditions. Fig. 6*E* plots ROCs for both fresh and weathered conditions. Data points for the fresh condition are based on the initial decision criteria from Fig. 6*A*. By contrast, data points for the weathered condition are based on the rescaled criteria from Fig. 6*D* (manifested in the olive curve as a bunching up of data points in the lower left corner of the ROC plot). The corresponding confidence–accuracy curve in Fig. 6*F* reveals that accuracy has been equalized across fresh and weathered conditions through rescaling of decision criteria on the basis of likelihood ratios. By this rescaling, the confidence–accuracy decoder succeeds in generalizing across increases in external noise (weathering).

In addition to these theoretical reasons to believe that the confidence–accuracy decoder might generalize across task difficulty, there exists empirical support for this claim from studies of eyewitness identification. Wixted and Wells (44), and subsequently Semmler et al. (45), reviewed the effects of task difficulty on the accuracy of lineup identifications. Task difficulty in this case is determined by “estimator variables,” which are stimulus conditions associated with the witnessed events, such as lighting, viewing distance, and duration (58). Depending upon their states, estimator variables may add significant noise to the eyewitness memory-based pattern comparison task—analogous to the external noise described above for forensic firearm examination. Consistent with likelihood-based rescaling of decision criteria across changes in task difficulty, the studies reviewed by these authors found that the confidence–accuracy relationship is remarkably consistent across differences in viewing duration, attentional focus, the presence of a weapon, the time until a lineup is performed, and other estimator variables that contribute noise to the decision process.

The only way that decision criteria can be appropriately rescaled based on likelihood ratios for *X* originating from *A* vs. *Ā* is, of course, if the examiner has acquired enough experience with relevant sensory conditions to detect changes in the underlying frequency distributions. Although this remains an empirical question ripe for investigation, it seems likely that seasoned forensic examiners, much like eyewitnesses (45), have gained such experience. If that proves to be the case, the personal equation strategy proposed here may hold great promise for improving the quality and utility of forensic pattern comparison decisions in the real world.

## Conclusions and Implications for the Courts

Absent truthful confession, our criminal justice system resorts to predictions about cause and culpability based on evidence found at the crime scene. That evidence often consists of patterned impressions discovered on the surfaces of objects, the source of which may reveal events and responsible actors. Our society has for many years relied heavily upon the human visual system for evaluating the origins of patterned evidence. In this capacity, the observer serves as an instrument for information measurement and classification. As for any such instrument, we’d like to know how well it works. The modern sciences of human information processing provide that knowledge. The human observer has empirically testable operating characteristics that establish its ability to resolve differences on the input, which can be used to assess the probability that an observer’s decision is correct.

The inconclusive option employed in pattern comparison disciplines neglects this science and supposes that performance measures are black and white. In doing so these disciplines flatten the probabilistic richness of sensory evidence and foster an illusion of certainty . As summarized above, that richness can be characterized empirically as continuous variation in the accuracy of a classification decision. With this nuanced information in hand, the trier of fact is in a better position to weigh the evidence.

All of this highlights a larger judicial and societal concern about the respective roles of forensic examiner and trier of fact. The examiner’s responsibility is to provide a conclusion qualified by an estimate of the probability that the conclusion is correct. The degree of accuracy that matters in the end is a decision to be made by the jury, not the forensic examiner. The established practice of defaulting on difficult problems, rather than using the fine sensitivity of the human visual system to mine the probabilistic richness of the evidence, permits the examiner to apply and propagate their own covert criteria for what constitutes meaningful information, thus shielding the fact finder from knowledge that could aid in deliberation. As jurist Learned Hand famously observed over a century ago, the expert is always at risk of invading the province of the jury and answering questions of fact according to their own understanding and biases: “Now the important thing and the only important thing to notice is that the expert has taken the jury's place if they believe him” (59).

## Data Availability

All study data are included in the main text.

## Footnotes

^*^In practice, the decision criterion is applied to the observer’s internal measure of the visual similarity between evidence and exemplar. To help conceptualize the larger source classification problem, I relate the decision variables and criteria back to sources *A* and *Ā* throughout this text, in line with the way source conclusions are commonly reported.

^†^The outcome of any decision criterion is also dependent on prior probabilities, or “base rates,” of stimulus *X* being produced by objects *A* and *Ā*. For simplicity, I assume here that sources *A* and *Ā* are equally probable causes of evidence *X*, manifested as symmetric frequency distributions in the illustrations that follow. The real world may, of course, present observers with different priors.

^‡^Proportion of correct identifications is computed here using true positive (TP) and false positive (FP) responses to obtain positive predictive value (PPV) = TP/(TP + FP), which is suitable for the special case in which prior probabilities of *A* and *Ā* are equal. The more general form, from Bayes rule, uses the prior odds ratio *P*(*A)* / *P*(*Ā)*, and the observed positive likelihood ratio (LR+ = TP rate/FP rate), to compute posterior odds = prior odds × LR+. Then, PPV = posterior odds/(posterior odds + 1). Similar logic applies to calculation of the proportion of correct eliminations.

^§^Pristine lineup conditions include lineup fillers chosen such that they similarly match the witness’ description of the perpetrator, making the lineup “fair,” and limits on biasing factors such as nonblinded lineup administration.

^#^The concept of likelihood-based scaling is fundamental to signal detection (27). The decision axis for any stimulus dimension *X* can be rescaled based on the ratio of likelihood that evidence *X* would be expected given *A* vs the likelihood that *X* would be expected given *Ā*.
